# Assessing the Predictive Efficacy of Lok Score in Identifying Esophageal Varices in Liver Cirrhosis Patients: A Cross-Sectional Study

**DOI:** 10.7759/cureus.48360

**Published:** 2023-11-06

**Authors:** Bilal Afzal Tarar, Abdullah Nadeem, Muhammad Zain Anees, Hassan Mumtaz, Maira Gardezi, Shanta Bai

**Affiliations:** 1 General Surgery, Northwick Park Hospital, London, GBR; 2 Internal Medicine, Dow University of Health Sciences, Karachi, PAK; 3 Gastroenterology, Bacha Khan Medical College, Mardan, PAK; 4 Urology, Guys & St Thomas Hospital, London, GBR; 5 General Practice, Surrey Docks Health Center, London, GBR; 6 Innovation, Implementation & Partnership Unit, Association for Social Development, Islamabad, PAK; 7 Gastroenterology, Faisalabad Medical University, Faisalabad, PAK; 8 Internal Medicine, Liaquat National Medical College and Hospital, Karachi, PAK

**Keywords:** lok score, non-invasive parameter, predictive tool, cirrhosis complications, gastro esophageal varices, liver cirrhosis

## Abstract

Introduction

Liver cirrhosis is a global health concern with various etiologies, leading to portal hypertension and gastroesophageal varices. Variceal bleeding, a severe complication of cirrhosis, necessitates early detection and intervention to reduce mortality. Endoscopic screening is the gold standard for varices detection but is invasive and expensive. This study evaluates the Lok Score, a non-invasive predictive tool, for identifying esophageal varices in patients with liver cirrhosis.

Materials and methods

A cross-sectional study involving 150 liver cirrhosis patients was conducted. The Lok score was calculated using specific parameters. Patient data, including age, gender, etiology of liver cirrhosis, Child-Pugh class, varices presence, and grades, were recorded. Statistical analysis was performed using IBM Corp. Released 2013. IBM SPSS Statistics for Windows, Version 22.0. Armonk, NY: IBM Corp., and diagnostic parameters for Lok Score were computed.

Results

The study demonstrates that the Lok score exhibits significant potential as a predictive tool for esophageal varices. The mean Lok score significantly differed between individuals with and without varices, suggesting a correlation between Lok score and varices presence. Higher Lok scores may indicate more advanced varices. Utilizing the Lok score in clinical practice could lead to timely interventions, improving patient outcomes.

Conclusion

The Lok score shows promise as a valuable predictive tool for esophageal varices in liver cirrhosis patients. Early identification using this non-invasive parameter can aid in risk stratification and guide appropriate management strategies. However, further validation and larger studies are needed to fully integrate the Lok score into clinical practice for the benefit of cirrhosis patients.

## Introduction

Cirrhosis of the liver is a prevalent illness that is widely observed across the globe. It is observed as a consequence of various factors, including but not limited to hepatitis B or C infection, obesity, non-alcoholic fatty liver disease, excessive alcohol intake, autoimmune disorders, cholestatic disorders, and excessive accumulation of iron or copper. The progression of cirrhosis occurs in normal liver tissue as a result of chronic inflammation, leading to fibrosis and the subsequent production of regenerating nodules. Portal hypertension is ultimately the result [1]. 

The occurrence of gastric varices is a consequence of portal hypertension, which arises from the establishment of collateral vessels connecting the portal vein and the superior vena cava through the azygos vein [2]. The rupture of gastroesophageal varices is associated with the occurrence of hematemesis, melena, or hematochezia, particularly in severe cases, and represents the most critical and potentially fatal consequence of cirrhosis [3].

There are other factors that can lead to life-threatening upper gastrointestinal bleeding (UGIB), such as peptic ulcer disease, angioectasias, Mallory-Weiss tear, erosive gastritis, erosive esophagitis, Dieulafoy lesion, and gastric cancer [4]. The findings of research conducted in Lahore, Pakistan, involving patients with upper gastrointestinal bleeding (58.6% females and 41.4% males) indicate that hepatitis C is the predominant etiology of upper gastrointestinal bleeding. Furthermore, the study highlights that hepatitis C-related upper gastrointestinal hemorrhage is more commonly associated with variceal bleeding than bleeding from ulcers [5].

A separate investigation conducted in the northern region of Pakistan involved a total of 490 participants, with 298 individuals (61%) identified as male and 192 individuals (39%) identified as female. The age group most frequently observed in cases of upper gastrointestinal bleeding is the elderly population, namely individuals aged 60 years and above (n=235, 47.9%). This is closely followed by individuals aged 40 to 59 years (n=174, 35.5%). Variceal bleeding was identified as the predominant etiology of upper gastrointestinal (GI) hemorrhage, accounting for the highest proportion (n=292, 59.5%). This was followed by ulcer bleeding (n=88, 18.0%) and gastric malignancy (n=28, 6%). In 82 instances, accounting for 17% of the cases, the etiology of gastrointestinal bleeding remained undetermined. The chi-square test revealed that variceal hemorrhage emerged as the most statistically significant factor [6].

Nevertheless, the majority of research undertaken on a global scale has consistently indicated that peptic ulcers are the predominant etiology of upper gastrointestinal bleeding (UGIB) [7]. A recent study conducted in Europe has provided confirmation of the prevalence of peptic ulcers, accounting for 44% of cases, and non-variceal ulcers, contributing to a total of 72% of cases of upper gastrointestinal bleeding (UGIB) [8].

The most reliable and widely accepted method for preventing variceal hemorrhage is endoscopic variceal screening, which is considered the gold standard in clinical practice. However, the surveillance of esophageal varices in cirrhotic patients through the utilization of endoscopy is a costly and invasive technique that may also cause discomfort to the patients [9]. Hence, in order to mitigate the psychological and financial strain, it is imperative to develop non-invasive indicators for the detection of esophageal varices [10]. In order to address the constraints prevalent in rural regions, where a significant number of health centers lack the necessary resources for performing esophagogastroduodenoscopy (EGD) and employing competent personnel, the utilization of non-invasive parameters might be considered as an alternative approach for predicting varices, hence obviating the need for EGD.

The Lok score is a valuable noninvasive diagnostic that exhibits a high level of accuracy in predicting the absence of major varices in individuals with cirrhosis. When the value exceeded 0.8, the positive predictive value for the prediction of big varices was 45.5%, the negative predictive value was 86.4%, and the diagnostic accuracy was 67.72%. In addition, the use of 10 also resulted in a higher number of averted endoscopies compared to the enlarged Baveno VI criteria while simultaneously maintaining a remarkably low miss rate with a negative predictive value above 99% [11].

The Lok Score cut-off value exceeding 0.9141 demonstrated a strong predictive capability in identifying big esophageal varices, with a sensitivity of 74.5%, a specificity of 72%, a positive predictive value of 84%, a negative predictive value of 58%, and an overall accuracy of 73.7% [3].

The core objective of this study was to discern the relationship between the Lok Score, a non-invasive parameter, and the presence of esophageal varices in patients diagnosed with liver cirrhosis. Esophageal varices, engorged veins in the lower esophagus, are common in individuals with liver cirrhosis and can lead to severe complications. Analyzing the predictive efficacy of the Lok score in identifying these varices is essential for effective treatment planning in such patients.

## Materials and methods

This cross-sectional study was conducted within the gastroenterology department of a tertiary care hospital over a six-month period. The sample size, determined to be 150 participants, was calculated using a validated sample size calculator. Non-probability consecutive sampling was utilized to select a diverse and representative sample.

Cirrhosis was delineated using precise ultrasonography criteria, which included the presence of uneven liver borders, splenomegaly exceeding 13.8 cm, or a portal vein diameter greater than 12 mm. These factors function as indications of the severity of cirrhosis. The Lok score, a pivotal statistic employed in this investigation, is derived by utilizing a specific formula:

Lok score: log odds = −5.56 - 0.0089 × platelet count (103/mm3) + 1.26 × (AST/ALT) + 5.27 × INR;

Lok = [exp (log odds)]/[1 + exp (log odds)] [12]

Sample selection

Inclusion criteria encompassed individuals aged 18 to 70, both genders, diagnosed with liver cirrhosis. Exclusion criteria included a history of variceal banding or sclerotherapy, beta-blocker use, or prior surgical procedures for varices (TIPSS). Hepatocellular carcinoma (HCC) patients were excluded to maintain specificity. By using the Rao Soft Sample size calculator, the sample size is 150, with a margin of error of 5%, a confidence interval of 95%, and a prevalence of 6.9% [13]. Nonprobability consecutive sampling technique was employed.

Data collection

Data collection commenced after obtaining ethical clearance. Crucial patient data, including age, gender, etiology of liver cirrhosis, CHILD class, presence of varices observed during endoscopy, and their grades, were meticulously recorded, forming the foundation for subsequent analysis.

Data analysis

The collected data underwent comprehensive analysis using IBM Corp. Released 2013. IBM SPSS Statistics for Windows, Version 22.0. Armonk, NY: IBM Corp., presenting results in frequencies and percentages. The Chi-square test was employed to evaluate the association between the Lok Score and esophageal varices. Key diagnostic parameters such as sensitivity, specificity, and predictive values were computed to assess the Lok Score's predictive accuracy in diagnosing significant esophageal varices during esophagogastroduodenoscopy.

## Results

**Figure 1:**
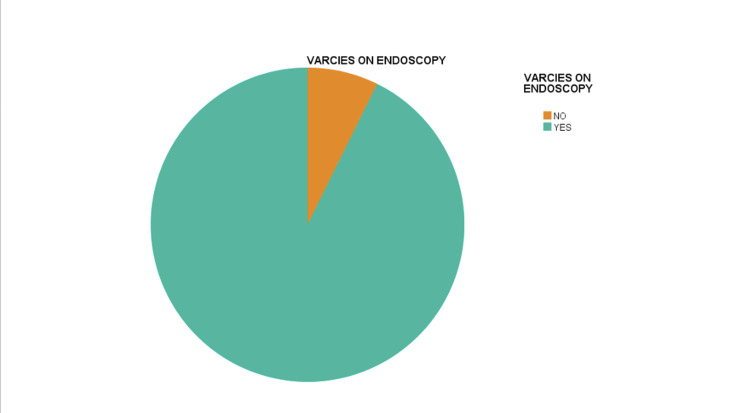
Presence of Varices on Endoscopy

The study encompassed a sample size of 150 individuals, with an average age of 52.54 years (standard deviation ± 8.88). In this study, the Lok score, an essential parameter under evaluation, exhibited an average value of 0.774 with a standard deviation of ± 0.24. The analysis of gender distribution indicated that 66% of the participants were male, while 34% were female. Regarding the etiology of liver cirrhosis, it is noteworthy that hepatitis C (HCV+) emerged as the primary factor responsible for 90.7% of cases. Non-B/C accounted for 4% of cases, while HBV+ and HBV+/HCV+ contributed to 3.3% and 2% of cases, respectively. In terms of clinical manifestation, a notable percentage of individuals exhibited no symptoms (76.0%), while 16.7% experienced symptoms, and 7.3% presented with bleeding. The distribution of patients among the Child-Pugh classes revealed that 22.7% were categorized as class A, 54.4% as class B, and 22.7% as class C. During the endoscopic examination, a significant proportion of patients (92.2%) exhibited varices, with high-grade varices being the most prevalent (33.3%), followed by medium-grade (35.3%) and low-grade varices (31.3%), as shown in Table 1 and Figure 1.

**Table 1:** Baseline Characteristics of Study Participants

Variables	Categories	Mean (n=150)	SD
Age (years) (mean± SD)	-	52.54	8.88
LOK score (mean± SD)	-	0.774	0.24
Variables	Categories	Frequency (n=150)	Percentage
Gender	Male	99	34%
	Female	51	66%
Etiology of liver cirrhosis	HBV+	5	3.3%
	HBV+/HCV+	3	2%
	HCV+	136	90.7%
	Non-B/C	6	4%
Presentation	Asymptomatic	114	76.0%
	Bleeder	11	7.3%
	Symptomatic	25	16.7%
Child Class	A	34	22.7%
	B	82	54.4%
	C	34	22.7%
Varices on endoscopy	Yes	139	92.2%
	No	11	7.3%
Grade of Varices	High	50	33.3%
	Low	47	31.3%
	Medium	53	35.3%

The examination of varices identified during endoscopic procedures yielded multiple noteworthy correlations. In relation to age, it was observed that the average age of individuals without varices was 43.18 years, but those with varices had an average age of 53.31 years, indicating a significant disparity in age between the two groups (p-value=0.725). In relation to the LOK score, a notable disparity was observed between persons without varices, who exhibited a mean LOK score of 0.3682±0.2, and those with varices, who displayed a higher mean LOK score of 0.8067±0.22 (p-value=0.02). There was no significant correlation seen between gender and the presence of varices, since the majority of males (91.9%) and females (94.1%) displayed varices (p-value=0.625). The etiology of liver cirrhosis was also examined, revealing no statistically significant correlation with varices. Nevertheless, an evident correlation was seen within the Child-Pugh classification, as a substantial proportion of people with varices were found to belong to either Child Class A (67.6%) or Child Class C (100%) groups (p-value=0.00), as shown in Table 2.

**Table 2:** Varices in Endoscopy and Associated Variables

Variables	Categories	No	Yes	P-value*
Age (years) (mean± SD)	-	43.18±7.4	53.31±8.57	0.725
LOK score (mean± SD)	-	0.3682±0.2	0.8067±0.22	0.02
Gender	Male	8 (8.1%)	91 (91.9%)	0.625
	Female	3 (5.9%)	48 (94.1%)	
Etiology of Liver Cirrhosis	HBV+	0 (0%)	5 (100%)	0.748
	HBV+/HCV+	0 (0%)	3 (100%)	
	HCV+	11 (8.1%)	125 (91.9%)	
	Non-B/C	0 (0%)	6 (100%)	
Child Class	A	11 (32.4%)	23 (67.6%)	0.00
	B	0 (0%)	82 (100%)	
	C	0 (0%)	34 (100%)	

This study aimed to evaluate the correlation between varices grade and several essential characteristics, namely age, LOK score, gender, etiology of liver cirrhosis, presentation, and child class. The average age for persons with high, low, and medium varices grades was around 54.60±7.9, 50.70±7.6, and 52.3±10.3 years, respectively. The results of the LOK score analysis revealed statistically significant variations across different grades of varices. The mean LOK scores were found to be 0.9114±0.56 for high-grade varices, 0.5698±0.28 for low-grade varices, and 0.827±0.14 for medium-grade varices (p-value=0.02). The analysis revealed a statistically significant relationship between gender distribution and varices grade (p-value=0.011). Specifically, high-grade varices were seen in 38.4% of males and 23.5% of females, low-grade varices were found in 23.2% of males and 47.1% of females, and medium-grade varices were present in 38.4% of males and 29.4% of females. Significant variations were identified (p-value=0.001) in the etiology of liver cirrhosis, with the hepatitis C virus (HCV) emerging as an important factor in the grading of varices. In addition, the presentation and child class exhibited statistically significant relationships, with p-values of 0.016 and <0.001, respectively, as shown in Table 3.

**Table 3:** Grade of Varices and Associated Variables

Variables	Categories	High	Low	Medium	P-value*
Age (years) (mean± SD)	-	54.60±7.9	50.70±7.6	52.3±10.3	0.725
LOK score (mean± SD)	-	0.9114±0.56	0.5698±0.28	0.827±0.14	0.02
Gender	Male	38 (38.4%)	23 (23.2%)	38 (38.4%)	0.011
	Female	12 (23.5%)	24 (47.1%)	15 (29.4%)	
Etiology of Liver Cirrhosis	HBV+	0 (0%)	5 (100%)	-	0.001
	HBV+/HCV+	0 (0%)	0 (0%)	3 (100%)	
	HCV+	48 (35.3%)	38 (27.9%)	50 (36.8%)	
	Non-B/C	2 (33.3%)	4 (66.7%)	0 (0%)	
Presentation	Asymptomatic	38 (33.3%)	29 (18.2%)	37 (32.5%)	0.016
	Bleeder	2 (18.2%)	0 (0%)	9 (81.8%)	
	Symptomatic	10 (40%)	8 (32%)	7 (28%)	
Child Class	A	3 (6%)	23 (48.9%)	8 (15.1%)	0.00
	B	26 (52.6%)	17 (23.2%)	39 (73.6%)	
	C	21 (42%)	7 (14.9%)	6 (11.3%)	

**Figure 2:**
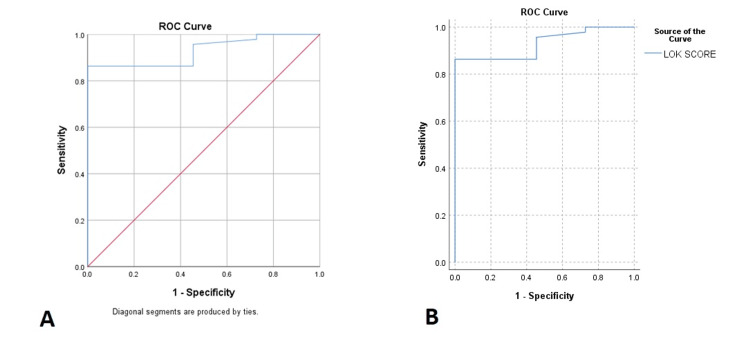
Receiver operating characteristic (ROC) curve for the presence of esophageal varices and the sensitivity and specificity of the LOK score A= ROC curve (left side), B = ROC analysis (right side)

At a specified threshold of 0.335 for the LOK score, the diagnostic test had a sensitivity of 93.5%, indicating its ability to correctly identify the presence of esophageal varices in 93.5% of instances. The specificity of the test was determined to be 54.5%, suggesting its ability to accurately identify individuals without esophageal varices around 54.5% of the time. The study findings indicate that the positive predictive value (PPV) of the test was 84.4%, indicating that the test accurately identified true cases 84.4% of the time when yielding a positive result. Conversely, the negative predictive value (NPV) demonstrated a rate of 75.0%, signifying its ability to accurately exclude the presence of esophageal varices in 75.0% of instances when the test yielded a negative result. The positive likelihood ratio (+LR) had a substantial value of 2.071%, indicating a notable elevation in the probability of having esophageal varices upon a positive test result. In contrast, the negative likelihood ratio (-LR) demonstrated a value of 0.104%, indicating a substantial reduction in the probability of esophageal varices when the test yielded a negative result, as shown in Table 4 and Figure 2.

**Table 4:** Diagnostic Performance Metrics for LOK Score at Cutoff Value 0.335

Cutoff Value	Sensitivity	Specificity	PPV	NPV	+LR	-LR	Diagnostic Accuracy
0.335	93.5%	54.5%	84.4%	75.0%	2.071	0.104	85.0%

## Discussion

Liver cirrhosis is an advanced phase of chronic liver disease characterized by the occurrence of significant consequences, including bleeding from varices in the esophagus and stomach, hypersplenism, ascites, hepatorenal syndrome, hepatopulmonary syndrome, and 14/19 syndrome. Certain individuals may develop liver cancer as their condition advances. Esophagogastric varices are a frequently occurring comorbidity, resulting in substantial mortality as a consequence of hemorrhaging on an annual basis. The rapid detection and assessment of the severity of esophageal and stomach varices play a crucial role in implementing preventative measures promptly, leading to a significant reduction in mortality rates [14-16]. Gastroscopy is widely recognized as the prevailing clinical approach for the screening of varices. However, there has been a recent movement in the academic community's attention towards non-invasive prognostic markers for the assessment of esophageal and gastric varices, as well as their severity.

In this study, the Lok score, which was carefully assessed, emerged as a potentially effective tool for predicting the existence and severity of esophageal varices in persons suffering from liver cirrhosis. The average Lok score among persons with varices (0.8067±0.22) is significantly higher than that of individuals without varices (0.3682 ± 0.2), indicating its potential as a predictive measure.

The notable disparity observed in the Lok score, when considering the presence or absence of varices, provides compelling evidence to support the potential utility of the Lok score as a reliable prognostic marker. Individuals with higher Lok scores exhibit a positive link between the Lok score and the existence of esophageal varices, suggesting that a higher Lok score may serve as an indicator of more advanced varices.

The consideration of clinical consequences is of utmost importance when utilizing the Lok score as a prediction tool for esophageal varices, particularly within the framework of liver cirrhosis. The rapid identification of varices has the potential to provide prompt therapies, including beta-blocker therapy, endoscopic variceal ligation, or transjugular intrahepatic portosystemic shunt (TIPS). These interventions have been shown to effectively mitigate the risk of variceal bleeding and enhance the overall results for patients. The Lok score has demonstrated promise as a beneficial noninvasive metric for evaluating the likelihood of esophageal varices, particularly in areas where there may be limited access to endoscopic expertise [17]. The proposed scoring system has the potential to function as a key benchmark for assessing the need for comprehensive screening and preventative interventions to reduce the likelihood of esophageal variceal bleeding in individuals diagnosed with liver cirrhosis. This is particularly relevant in regions where advanced endoscopic capabilities are not readily available. As a result, the utilization of this method can assist in the stratification of persons who are at risk, providing a non-intrusive option for the evaluation of esophageal varices within the context of liver cirrhosis [18].

However, it is crucial to do additional validation and refinement of the Lok score in order to establish its efficacy as a predictive tool. In order to thoroughly evaluate the prediction accuracy of the Lok score and its possible consequences for clinical practice, it is imperative to conduct longitudinal studies that track the course of varices over time, utilizing a larger and more diverse patient group.

Furthermore, given the complex and diverse nature of liver cirrhosis and its various causes, it would be advantageous to carry out research that is categorized according to different etiological aspects. This approach would allow for a more detailed assessment of the predictive capability of the Lok score. This study aims to investigate if the prediction capacity of the Lok score varies depending on the etiology of liver cirrhosis.

Limitations

The study presents valuable insights into the potential use of the Lok score as a non-invasive predictive tool for identifying esophageal varices in liver cirrhosis patients. However, there are some limitations that should be considered when interpreting the results.

First, the study employed non-probability consecutive sampling, which can introduce selection bias, potentially limiting the generalizability of the findings. This sampling method may not represent the broader population of interest, and the study's findings may be specific to the patient population encountered during the study period. Additionally, the relatively small sample size and the single-center nature of the study may impact the statistical power and generalizability of the results. Larger, multicenter studies with more diverse patient populations are needed to strengthen the external validity of the findings. Furthermore, the cross-sectional design provides a snapshot of data at a specific point in time, and future research could benefit from longitudinal studies to better understand trends and changes over time. Finally, the study's exclusion criteria, including the omission of certain patient groups, may have influenced the results and should be considered when interpreting the findings.

Despite these limitations, this research provides valuable insights into the predictive efficacy of the Lok Score for identifying esophageal varices in liver cirrhosis patients and underscores the need for further investigations to refine and validate this non-invasive parameter in clinical practice.

## Conclusions

In conclusion, the comparative analysis of Lok score, a noninvasive parameter known for its efficacy in predicting the size of esophageal varices in liver cirrhosis patients, revealed significant distinctions between cohorts with large and small esophageal varices as ascertained through endoscopic examination. This investigation underscores the potential integration of Lok score with adjunctive noninvasive methodologies, such as fibroscan or elastography, targeting the assessment of portal hypertension. The envisioned synthesis of these noninvasive modalities presents a promising avenue for the construction of a comprehensive algorithm harmonizing various parameters to predict the presence, magnitude, and absence of esophageal varices in liver cirrhosis patients. Ongoing research endeavors aimed at validating and refining this integrated approach are imperative to optimize its clinical utility, aiding in precise prognostication and tailored therapeutic strategies for individuals grappling with liver cirrhosis.
